# Commentary on: “Delayed necrostatin-1s infusion attenuates cystic white matter injury in preterm fetal sheep.”

**DOI:** 10.1016/j.neurot.2025.e00810

**Published:** 2025-11-27

**Authors:** Stephen A. Back

**Affiliations:** Departments of Pediatrics and Neurology, Oregon Health & Science University, Portland, OR 97239, USA

**Keywords:** Periventricular leukomalacia, Cerebral palsy, White matter, Prematurity, Necroptosis, Necrosis

Preterm birth is a major global health problem that carries a significant risk for life-long neurodevelopmental disabilities. Rates of prematurity worldwide are not declining and roughly range between 5 and 15 ​% [1]. Of all live births in the U.S., 1 in 10 are born preterm. Although neonatal care advances have led to significant improvements in viability of very low birth weight (VLBW) infants, improved survival has been accompanied by growing numbers of pre-term survivors with chronic neurological disabilities that include the spastic motor deficits of cerebral palsy (CP) as well as a broad spectrum of cognitive and learning disabilities.

Cerebral white matter injury (WMI) is the leading cause of brain injury in preterm infants [2]. The most severely compromised infants typically sustain cystic WMI, otherwise known as periventricular leukomalacia (PVL). PVL was for decades the dominant form of cerebral WMI, reported in greater than 40 ​% of preterm survivors [3]. Although PVL has markedly declined to ∼5 ​% of all cases of WMI, it remains a devastating condition for which no effective therapies exist to prevent or reverse brain injury. It should be noted that most VLBW infants currently sustain diffuse WMI that involves selective loss of oligodendrocyte progenitors that predominate in the cerebral white matter at this time in development [4]. WMI, thus, disrupts the normal process of developmental myelination. Although overt loss of neurons is not a significant feature of isolated forms of diffuse WMI, neuronal dysmaturation appears to be a significant contributor to the persistent neurobehavioral sequelae that accompany diffuse WMI [5].

By contrast, the cystic nature of PVL arises due to white matter necrosis that triggers diffuse loss of both neuroglia and axons [2]. In severe cases, PVL progresses to macroscopic lesions ranging from ∼1 to 6 ​mm in diameter that localize preferentially to the periventricular and deep white matter. In extreme cases, the necrosis may extend into superficial white matter and evolve into confluent multi-cystic lesions. Axonal degeneration manifests as ventriculomegaly and impaired growth of cortical or subcortical gray matter structures due to secondary retrograde neuronal loss [2].

While macroscopic cystic lesions are readily identified on cranial MRI, it is important to note that the spectrum of white matter necrosis also involves microscopic lesions (i.e., microcysts). The incidence and clinical significance of microcysts remain unclear, since they are too small for detection by clinical MRI. Microcysts were detected in >30 ​% of cases in one autopsy study but comprised only ∼1–5 ​% of the total burden of WMI [6]. Nevertheless, it seems likely that microcysts range from clinically silent to functionally significant lesions depending upon their number and distribution.

Multiple studies in human and preterm fetal sheep support that the severity of WMI is related to a combination of vascular anatomic immaturity, disturbances in cerebrovascular autoregulation and a variety of metabolic and neuroinflammatory factors that may predispose to more severe energy failure in regions of focal white matter necrosis [7]. Notably, the distribution of microcysts is similar to that of cystic necrosis, which further suggests a similar hypoxic-ischemic origin.

Given the rapidity and severity of necrotic cellular degeneration, it has been widely assumed that early interventions would be unlikely to have a significant impact on the progression of white matter necrosis. However, it is important to note that early white matter necrosis is followed by subsequent stages of cellular inflammatory responses, which over weeks lead to clearance of cellular debris by infiltrating macrophages and the formation of astroglial scars that wall off lesions [2].

In this issue of *Neurotherapeutics*, Lear and colleagues tested the intriguing possibility that the progression of necrotic WMI triggered by hypoxia-ischemia might be disrupted during the delayed phase of slowly evolving programmed necrosis to produce milder and less extensive WMI [8]. To test this hypothesis, they employed a clinically relevant large preclinical animal model in which preterm fetal sheep sustained systemic hypoxia-ischemia via 25 ​min of umbilical cord occlusion (UCO). This well-established UCO model delivers whole body asphyxia that mimics human fetal pathophysiological responses to *in utero* UCO. A significant strength is the unique tolerance of the pregnant ewe to *in utero* instrumentation that permits real-time continuous fetal monitoring of fetal heart rate, blood pressure, EEG and continual sampling of fetal blood gases and metabolic responses [9]. The large size of the preterm fetal head at ∼0.65 gestation also permits intracerebroventricular (i.c.v.) infusions of neuroprotective agents.

Since the pathogenesis of PVL involves necrosis, the authors tested the hypothesis that PVL might be mediated by a form of programmed necrosis (necroptosis) amenable to delayed intervention with an inhibitor of this pathway. They chose to test necrostatin-1s (Nec-1s) since previous studies found that Nec-1s reduced the magnitude of hypoxic-ischemic cerebral injury in near term newborn mice [10]. Nec-1 targets the pro-necroptotic protein RIP1, an upstream target of the necroptosis pathway, and thereby blocks formation of the necrosome [11]. Nec-1s or vehicle was infused i.c.v. at 3, 8 and 13 days after UCO to test whether delayed treatment could reduce the burden of necrosis. Vehicle treated controls displayed key features of PVL including white matter atrophy, ventriculomegaly and cystic WMI. Although human WMI particularly targets frontal and parietal-occipital white matter, the WMI pattern of preterm fetal sheep was reproducibly temporal and parietal. The reproducibility of the lesion patterns was critical to quantification of the response to Nec-1s. The authors found that Nec-1s demonstrated delayed protection against necrotic WMI in some white matter tracts. Despite the primary focus of this study on WMI, reduced gray matter atrophy was also found in cerebral cortex, thalamus and hippocampus. Notably, temporal lobe, but not parietal white matter lesions, had decreased atrophy and microgliosis. An increased density of myelinating oligodendrocytes and an apparent increase in myelination was consistent with the predilection for oligodendrocyte progenitors to degenerate via necrosis. On a cautionary note, the authors also found that Nec-1s treatment augmented the fraction of apoptotic cells in essentially all white matter tracts studied, which suggested that some delayed cell death was routed from necrosis to apoptosis. This study, thus, provides important support in a large preclinical animal model that delayed treatment of necrotic WMI could be beneficial, perhaps by disrupting necrosis-triggered inflammatory cascades, as suggested by another recent study from this group with Etanercept, which blocks the action of tumor necrosis factor [12].

This study raises several provocative questions. Were the disparate regional responses to Nec-1s related to differences in the timing of injury such that there may be benefit from delivery of an earlier dose within hours after HIE? Defining the evolution of early injury by diffusion-weighted MRI could provide valuable information about regional differences in the onset of necrosis, but would need to be weighed against the challenges of *in utero* MRI studies or ex vivo high field imaging [13].

Future studies are warranted to further define the cell death pathways targeted and disrupted by Nec-1s. Could inhibitors of apoptosis be combined with necrostatin-1 for more effective recovery of viable white matter? Given that oligodendrocyte progenitors are iron-rich cells susceptible to oxidative stress from glutathione depletion [14], may there also be a shift to enhanced ferroptosis? Could there also be a role for combination therapies with Nec-1s and Etanercept to further reduce inflammatory mediated sequelae of programmed necrosis by targeting two distinct parts of the necroptosis pathway?

From a clinical standpoint, it would be valuable to address whether the burden of microscopic necrosis is also reduced by Nec-1s. Since microcysts persist even with milder contemporary forms of diffuse preterm WMI, it will be important to determine whether there is a role for Nec-1s in preventing these milder but potentially significant forms of white matter necrosis. Microcysts might be more readily detected by reducing the severity of the model, as previously demonstrated in preterm fetal sheep [13]. Since the impact of PVL often extends to secondary gray matter injury [15], quantitative studies would be valuable to define regional differences in the impact of necrostatin-1 on neuronal loss in cortical versus subcortical gray matter structures. Since WMI commonly occurs in the first days of life during intensive care, it will also be important to determine whether delayed delivery of Nec-1s has benefit in the setting of preterm WMI that is acquired postnatally. Given that long-term outcome studies are extremely costly in human preterm survivors, the preterm fetal sheep offers some attractive options for accelerated evaluation of long-term functional benefits. It is feasible in fetal sheep to conduct *in utero* functional studies of axonal integrity in some white matter tracts with somatosensory and visual evoked potentials [9]. A number of behavioral studies are also feasible in juvenile or adult sheep including cognitive and memory testing [16,17].

Developing therapies to significantly reduce the impact of the most severe forms of white matter injury is likely to be fraught with significant challenges. Brain injury in preterm infants is a moving target that occurs against a complex backdrop of numerous intricately timed and inter-related neurodevelopmental events. As this study illustrates, at any given time, not all brain regions will respond equivalently to a given therapy. Moreover, injured cells can progress along more than one cell death pathway, as illustrated here by the shift from necrosis to apoptosis. Nevertheless, the present study represents a significant advance in that it provides unexpected new directions to develop rational therapies for some of our most severely affected preterm neonates.

## Author contributions

Commentary written by sole author.

## Declaration of competing interest

The authors declare the following financial interests/personal relationships which may be considered as potential competing interests: Stephen Back reports financial support was provided by National Institutes of Health. If there are other authors, they declare that they have no known competing financial interests or personal relationships that could have appeared to influence the work reported in this paper.
